# Does supportive legislation guarantee access to pregnancy termination and postabortion care services? Findings from a facility census in Central Province, Zambia

**DOI:** 10.1136/bmjgh-2018-000897

**Published:** 2018-09-03

**Authors:** Jenny A Cresswell, Onikepe O Owolabi, Nachela Chelwa, Mardieh L Dennis, Sabine Gabrysch, Bellington Vwalika, Mike Mbizvo, Veronique Filippi, Oona M R Campbell

**Affiliations:** 1 Department of Infectious Disease Epidemiology, London School of Hygiene & Tropical Medicine, London, UK; 2 Population Council, Lusaka, Zambia; 3 Institute of Public Health, University of Heidelberg, Heidelberg, Germany; 4 School of Medicine, University of Zambia, Lusaka, Zambia

**Keywords:** epidemiology, health services research, maternal health, public health

## Abstract

**Introduction:**

Zambia is one of the few countries in Africa to permit termination of pregnancy (TOP) on a wide range of grounds. However, substantial barriers remain to TOP and postabortion care (PAC).

**Methods:**

We conducted a census of 153 facilities between March and May 2016. We defined facilities according to whether they met basic and/or comprehensive signal functions criteria for TOP and PAC. We linked our facility data to census data to estimate geographic accessibility under different policy scenarios.

**Results:**

Overall, 16% of facilities reported they had performed a TOP and 39% performed a PAC in the last year. Facilities were twice as likely to use medical methods for TOP compared with surgical methods, and four times more likely for PAC. Considerably more facilities had performed TOP or PAC than met the basic or comprehensive signal functions criteria, indicating services were being performed in facilities below essential quality standards. Under current Zambian law for non-emergency scenarios, 21% of women in Central Province lived within 15 km of a facility with basic capability to provide TOP; if midlevel providers were trained to provide TOP, this would increase to 36%.

**Conclusion:**

A supportive legislative framework is essential, but not in itself sufficient, for adequate access to services. Training midlevel providers, in line with WHO guidance, and ensuring equipment is available in primary care can increase accessibility of TOP and PAC. While both medical and surgical methods need to be available, medical abortion is a safe and effective method that can be provided in low-resource settings.

**Key questions**

**What is already known?**

- Zambia is one of the few countries in Africa to allow termination of pregnancy (TOP) on a wide range of legal grounds; however, unsafe abortion is high as multiple barriers to care remain.

**What are the new findings?**

- Geographic accessibility to abortion services at an acceptable level of quality in Central Province, Zambia, is low, despite the relatively unrestrictive legal framework.
- Allowing midlevel providers to provide abortion services, in line with WHO guidelines, would increase geographic accessibility in the population without the need for substantial additional resources, using existing primary health services.

**What do the new findings imply?**

- Implementing programmes to train midlevel providers, in line with WHO guidance, and ensuring equipment is available in primary care can increase accessibility of TOP and postabortion care.

## Introduction

An estimated 8.3 million induced abortions took place in Africa annually between 2010 and 2014^1^; less than 10% of which were a safe termination of pregnancy (TOP) following WHO guidelines.^2–4^ Safe TOP can reduce maternal mortality because they reduce recourse to unsafe abortion and because contraceptive counselling can help prevent future unwanted pregnancies. While all abortions, either induced or spontaneous, may need postabortion care (PAC) to treat arising complications, the probability of requiring PAC is greatest for unsafe abortions.

Zambia is one of the few countries in Africa to permit legal TOP on a wide range of grounds. The *Termination of Pregnancy Act* of 1972 states that an abortion may take place if the continuation of the pregnancy involves a risk to the pregnant woman’s life, physical or mental health; a risk to the health of any existing children; or if there is a substantial risk of birth abnormalities.^5 6^ Three doctors’ signatures are required, although this is waived if one doctor believes TOP is immediately necessary to protect the woman’s health. Despite this, the law is still a matter of contention, and in recent years, there have been attempts to amend the Zambian constitution with a draft article that defines life as beginning at conception^7 8^; the political environment remains sensitive.

Zambia has a high burden of abortion-related morbidity and mortality due to the multiple barriers that exist in accessing safe, legal services: the abortion-related near-miss rate across Central, Copperbelt and Lusaka provinces is 72 per 100 000 women, and the abortion-related near-miss ratio is 450 per 100 000 live births.^9^ Zambia identifies as a country with a strong Christian culture.^10^ Women in Zambia are generally unaware that a TOP can be legally obtained in a wide range of circumstances, which delays and limits care-seeking.^11^ Economic and social costs are high, and the procedure is strongly socially stigmatised.^12^ Women must often travel long distances to reach a health facility that can provide the procedure. There is a shortage of health providers: in Central Province, where our study took place, there is one medical doctor for every 111 648 people.^13^ Within health facilities, conscientious objection, lack of training, high staff turnover and stigma all present further barriers to care.^14 15^

While the many barriers to accessing abortion services in legally restrictive settings are relatively well documented, much less is known about the levels of provision and quality of care within health systems in these settings. The WHO has used the signal functions approach to document provision of emergency obstetric care for several decades,^16^ and recently an extension of this approach has been proposed for abortion services.^17^ The advantages of using a signal functions approach are: the ability to assess health system capabilities, make comparisons across time and place and describe service provision in terms of equity while requiring relatively little data and avoiding placing undue burden on data collection systems.^17^

The aim of this study was to characterise the inputs for service provision for TOP and PAC services in Central Province, Zambia, in 2016 using the signal functions approach. In addition, we had the following objectives: (1) to evaluate time trends, where data permitted; (2) to estimate health facilities’ potential capability to provide services under three different policy assumptions; and (3) to examine geographic access to TOP and PAC services by linking our facility data with the last population census.

## Methods

### Data sources

#### Setting

Central Province is one of ten provinces in Zambia, with a population of 1.3 million.^18^ It is the third largest province by area and midranking in terms of population density. In 2012, the provincial and district boundaries of Central Province changed; throughout this paper, we use ‘Central Province’ to refer to the area covered by the post-2012 boundaries.

#### Health facility census (2016)

We conducted a census of health facilities in Central Province between March and May 2016. Our sampling frame was a list of 203 health facilities obtained from the Ministry of Health (MOH), of which 191 were eligible for our study (nine were no longer in Central Province due to the administrative boundary change; two facilities had not yet opened; one facility was in a male-only prison). We identified 26 health facilities missing from the original list via key informants. Thus, 217 facilities were eligible for inclusion in our census. Of these, 193 facilities agreed to participate (89% response rate); most of the refusals were military facilities (n=11) with two government facility refusals, and 11 facilities were inaccessible due to the rains. We had good representation across both public and private sectors. In this paper, we present data from the 153 hospitals and health centres. We excluded health posts (n=40) because they are not expected to provide TOP; while they can theoretically provide PAC, the vast majority are not open 24/7 nor do they have the requisite staffing. A map of facilities in this study is available as online supplementary appendix A.

10.1136/bmjgh-2018-000897.supp1 Supplementary data

Each facility was visited by a trained interviewer who used a structured questionnaire to collect information on staffing and opening hours; the clinical functions that the facility could perform and those that were performed in the last year; and equipment and commodity availability and observed functionality. The questionnaire was pretested in four facilities outside of Central Province prior to fieldwork. Use of handheld tablet computers allowed for ongoing data monitoring; if any issue was identified, then the project coordinator contacted the facility or district health office to verify the response. The questionnaire is available to view through the London School of Hygiene & Tropical Medicine Data Compass repository (see Data sharing statement below).

#### Health facility census (2005)

We used secondary data from a 2005 national facility census with funding from the Japan International Cooperation Agency.^19^ It covered 1421 facilities in Zambia, 144 of which were in Central Province, comprising all public, mission and non-governmental organisation facilities and some larger private-for-profit facilities. The census asked about infrastructure, utilities, equipment, service delivery and human resources. It was not designed to look specifically at abortion services; however, questions on staffing and family planning were comparable with those used in our study.

#### Population data

Population data were obtained from the 2010 Census of Population and Housing.^18^ The census lists a population count at the ward level, disaggregated by sex. We applied the female-specific district-level population growth rates to estimate the female population living in each ward in 2016. The growth rates are published within the census and are based on annualised growth for the period 2000–2010. Geographic shapefiles describing the 2010 administrative boundaries at the ward level^20^ were edited to map the boundaries of Central Province as of 2016.

### Analysis

Capability refers to the ability to perform a given clinical service to a minimum acceptable level of quality; it does not necessarily mean that the service has been provided. Conversely, a service could have been provided, but the facility not deemed to have the appropriate capability if a key indicator of quality or appropriate content was not met. We defined two levels of TOP and PAC capability: basic and comprehensive, corresponding to the care that a health centre or a hospital, respectively, would typically be expected to be capable of providing (table 1). These criteria have been described previously^17^; however, we modified them as follows in this study: (1) we developed more specific criteria regarding the trimester of pregnancy, and (2) we removed the requirement for a means of communication or transport availability as we felt that our instruments had not adequately captured the construct of referral capability.

**Table 1:** Definitions of basic and comprehensive capability to provide TOP and PAC services

Basic capability to provide TOP	Facility states that it would be able to provide medical and/or surgical TOP during the first trimester. Facility has at least three different methods of family planning (including condoms) available at time of survey. Family planning services are offered at least 1 day/week. At least one health professional present at time of survey. At least one doctor registered at the facility.
Comprehensive capability to provide TOP	Facility states that it would be able to provide medical and/or surgical TOP during both the first and second trimesters. Facility has at least three different methods of family planning (including condoms) available at time of survey. Facility has at least one long-acting reversible method of family planning available at the time of the survey (intrauterine device or implant). Family planning services are offered at least 1 day/week. At least one health professional present at time of survey. At least one doctor registered at the facility.
Basic capability to provide PAC	Facility states that it would be able to provide medical and/or surgical PAC during the first trimester. Facility is open 24 hours/7 days. Facility can give parenteral antibiotics. Facility can give uterotonics. Facility can give intravenous fluids. Facility has at least three different methods of family planning (including condoms) available at time of survey. Family planning services are offered 7 days/week. At least one health professional present at time of survey. At least three health professionals registered at the facility.
Comprehensive capability to provide PAC	Facility states that it would be able to provide medical and/or surgical PAC during both the first and second trimesters. Facility is open 24 hours/7 days. Facility can give parenteral antibiotics. Facility can give uterotonics. Facility can give intravenous fluids. Facility can give a blood transfusion. Facility can conduct emergency surgery (laparotomy, laparoscopy and/or hysterectomy). Facility has at least three different methods of family planning (including condoms) available at time of survey. Facility has at least one long-acting reversible method of family planning available at the time of the survey (IUD or implant). Family planning services are offered 7 days/week. At least one doctor present at time of survey. At least three doctors registered at the facility.

PAC, postabortion care; TOP, termination of pregnancy.

Data were cleaned, and descriptive statistics were calculated using Stata SE V.14.2. We reported the percentage of facilities that met each criterion, stratified by facility level. We had very few missing data. One facility was missing information about the availability of IUDs, and one facility was missing data about the availability of implants; in both cases, we coded these as ‘not available’. ‘Health professional’ was defined as a medical doctor, clinical officer, midwife or nurse.

We estimated the geographic accessibility of the population to facilities with capability to provide basic and comprehensive TOP services under three different scenarios. These different scenarios do not affect the classification of capability to provide PAC, so we only estimated this once.

#### Current Zambian law under 1972 TOP Act: non-emergency scenario

We defined a facility as meeting this criterion if at least three doctors were registered. In Zambia, a doctor is considered registered if they are on the full register of the Health Professions Council and can work independently.

#### Current Zambian law under 1972 TOP Act: emergency scenario

We required a facility to have at least one doctor registered to meet this criterion.

#### WHO guidelines allowing midlevel providers to provide TOP services

WHO guidelines state that ‘abortion care can be safely provided by any properly trained health care provider including midlevel providers’.^3^ Zambian MOH guidelines on reducing unsafe abortion also state that, with appropriate training, healthcare providers who are not doctors can provide TOPs during the first trimester,^6^ although this is not widely implemented.

Women living within 5 km and 15 km of a facility meeting the above criteria were estimated using ArcGIS 10.3. First, population density per ward was calculated by dividing the population count by the area covered. Geographic positioning system coordinates located health facilities within wards. A circle with a radius of 5 km or 15 km was drawn around the position of each facility, and the population living within this zone was estimated by calculating the proportion of each ward lying inside or outside the circle. We repeated this analysis for women living within urban wards (defined as population density >200 women per square kilometre) and rural wards.

## Results

Between 2005 and 2016, the number of health centres and hospitals in Central Province increased from 125 to 153. However, the proportion of facilities with minimum staffing did not improve; in 2005, 88% of facilities had a health professional present at the survey, compared with 84% in 2016. Availability of contraceptive commodities improved between 2005 and 2016 (table 2). There was substantial improvement in availability of long-acting reversible methods such as implants and IUDs.

**Table 2:** Changes in family planning availability and facility staffing in central province between 2005 and 2016 by level of facility

	2005		2016	
	Hospitals n=10, %	Health centres n=115, %	Hospitals n=10, %	Health centres n=143, %
Family planning availability				
Male condom	50	94	90	93
Female condom	20	17	70	43
Combined contraceptive pill/oral contraceptives	30	93	100	80
Progesterone-only contraceptive pill	30	77	80	66
Combined injectable	30	92	70	44
Progesterone-only injectable			100	90
Implant	20	1	90	57
IUD	30	3	70	31
Emergency contraception	0	13	80	31
Any method of family planning	60	96	100	97
At least three different method types* available, including condoms	30	94	90	87
At least one long-acting reversible method available	40	3	90	58
Family planning available 1+ day/week	50	97	90	97
Family planning available 7 days/week	20	23	40	17
Facility staffing				
Has 1+ health professional present	100	87	100	83
Has 3+ health professionals registered	100	42	100	41
Has 1+ doctor present	80	4	90	12
Has 3+ doctors registered	20	0	70	2

*Combined and/or progesterone-only of a method defined as one type; male and/or female condom defined as one type.

In 2016, 16% of facilities (70% of hospitals, 13% of health centres) had performed TOP during last 12 months (table 3). More than twice as many facilities had performed TOP in the first trimester compared with the second trimester. Similarly, more than twice as many facilities had performed TOP by medical versus surgical methods. Thirty-nine per cent of facilities (100% of hospitals, 35% of health centres) had performed PAC in the same period. Around four times more facilities had performed PAC by medical versus surgical methods.

**Table 3:** Facility performance and availability of abortion-related signal functions in Central Province in 2016 by level of facility

	Hospitals n=10, %	Health centres n=143, %	Total n=153, %
Facilities that have performed a procedure within last 12 months			
Medical termination of pregnancy	80	12	16
Surgical termination of pregnancy	40	3	6
First trimester termination of pregnancy (medical or surgical)	80	12	16
Second trimester termination of pregnancy (medical or surgical)	40	3	6
Any termination of pregnancy (medical or surgical)	70	13	16
Medical postabortion care	90	36	39
Surgical postabortion care	70	5	9
First trimester postabortion care (medical or surgical)	90	26	30
Second trimester postabortion care (medical or surgical)	80	11	16
Any postabortion care (medical or surgical)	100	35	39
Facilities that report it would be able to perform procedure			
First trimester termination of pregnancy (medical or surgical)	80	13	12
Second trimester termination of pregnancy (medical or surgical)	70	3	8
First trimester postabortion care (medical or surgical)	100	41	54
Second trimester postabortion care (medical or surgical)	80	22	26
Capability to carry out specific clinical procedures			
Give intravenous fluids	100	99	99
Give parenteral antibiotics	100	98	98
Give uterotonics	100	87	88
Give a blood transfusion	100	2	9
Conduct a laparotomy, laparoscopy and/or hysterectomy	80	2	7
Family planning availability			
Male condom	90	93	93
Female condom	70	43	45
Combined contraceptive pill/oral contraceptives	100	80	82
Progesterone-only contraceptive pill	80	66	69
Combined injectable	70	44	46
Progesterone-only injectable	100	90	91
Implant	90	57	59
IUD	70	31	34
Emergency contraception	80	31	33
At least three different method types* available, including condoms	90	87	87
At least one long-acting reversible method available	90	58	60
Family planning available 1+ day/week	90	97	96
Family planning available 7 days/week	40	17	18
Facility staffing			
Has 1+ health professional present	100	83	84
Has 3+ health professionals registered	100	41	45
Has 1+ doctor present	90	12	17
Has 3+ doctors registered	70	2	7
Facility is open 24 hours a day, 7 days per week	90	30	34

*Combined and/or progesterone-only of a method defined as one type; male and/or female condom defined as one type.

The ability to carry out clinical procedures was associated with facility level. All hospitals could give intravenous fluids, parenteral antibiotics, uterotonics and blood transfusions, while most could conduct a laparotomy, laparoscopy or hysterectomy. In contrast, while nearly all health centres could give intravenous fluids, parenteral antibiotics or uterotonics, as expected, few health centres could give a blood transfusion or conduct surgery.

Most facilities had at least three methods of contraception available, and nearly all facilities were able to provide family planning at least once a week. Availability of long-acting reversible methods was associated with facility level. Only 40% of hospitals and 17% of health centres could provide family planning every day. Emergency contraception was available in one-third of facilities.

All hospitals and 83% of health centres had at least one health professional present in the facility at the time of the survey; 90% of hospitals and 12% of health facilities had a doctor present. Most hospitals and around a third of health centres were open 24/7, although two health centres that reported being open 24/7 did not have a health professional present during the survey.

Considerably more facilities had performed TOP or PAC in the last 12 months than met the signal functions criteria for basic capability (table 4). For TOP services, just 6% of facilities met the basic criteria, with 5% of facilities meeting comprehensive criteria under the three doctors requirement. If midlevel providers routinely provided TOP, 16% of facilities would meet the basic criteria and 7% would meet the comprehensive criteria. For PAC, 4% of facilities met the basic criteria, while only 2% of facilities met the comprehensive criteria.

**Table 4:** Percentage of facilities achieving basic and/or comprehensive capability to carry out TOP services and/or PAC in Central Province under alternative scenarios by level of facility

	Hospitals n=10, %	Health centres n=143, %	Total n=153, %
Current law under 1972 TOP Act, non-emergency scenario: requirement for three doctors to be registered			
Basic capability to provide TOP	60	1	5
Comprehensive capability to provide TOP	50	1	4
Current law under 1972 TOP Act, emergency scenario: requirement for one doctor to be registered			
Basic capability to provide TOP	70	1	6
Comprehensive capability to provide TOP	60	1	5
WHO guidelines allowing midlevel providers to provide TOP services			
Basic capability to provide TOP	70	12	16
Comprehensive capability to provide TOP	60	3	7
Provision of PAC services			
Basic capability to provide PAC	30	2	4
Comprehensive capability to provide PAC	20	1	2

PAC, postabortion care; TOP, termination of pregnancy.

Under the three doctors requirement, 21% of women lived within 15 km of a facility with basic or comprehensive capability to perform TOP services with strong urban–rural inequity (9% of rural women; 100% of urban women) (table 5). If midlevel providers provided TOP, 36% of women would live within 15 km of a facility with basic capability (26% of rural women) and 26% within 15 km of a facility with comprehensive capability (15% of rural women). Around one in four women lived within 15 km of a facility with basic capability to provide PAC; 19% lived within 15 km of a facility with comprehensive capability.

**Table 5:** Proximity of the female population to facilities with basic and/or comprehensive capability to carry out TOP services and/or PAC in Central Province under alternative scenarios

	Urban		Rural		Overall	
	Female population living within 5 km, %	Female population living within 15 km, %	Female population living within 5 km, %	Female population living within 15 km, %	Female population living within 5 km, %	Female population living within 15 km, %
Current law under 1972 TOP Act, non-emergency scenario: requirement for three doctors to be registered						
Basic capability to provide TOP	86	100	2	9	13	21
Comprehensive capability to provide TOP	77	100	2	9	12	21
Current law under 1972 TOP Act, emergency scenario: requirement for one doctor to be registered						
Basic capability to provide TOP	89	100	2	12	13	24
Comprehensive capability to provide TOP	77	100	2	12	12	24
WHO guidelines allowing midlevel providers to provide TOP services						
Basic capability to provide TOP	100	100	4	26	17	36
Comprehensive capability to provide TOP	77	100	3	15	12	26
Provision of PAC services						
Basic capability to provide PAC	89	100	2	12	14	24
Comprehensive capability to provide PAC	77	100	1	7	11	19
Facility has provided TOP in last 12 months	100	100	4	26	17	36
Facility has provided PAC in last 12 months	100	100	9	53	21	59

PAC, postabortion care; TOP, termination of pregnancy.

## Discussion

Despite Zambia’s relatively non-restrictive laws, geographic accessibility to safe abortion services in Central Province is very low. Zambia, and in particular Central Province, has a major shortage of health workers^13^ and the current legal requirement for three doctors’ signatures represents a substantial barrier to accessing care. Stigma, both in the community and among providers, is another key factor.^11 14^ Other studies have used similar criteria to measure the functioning of facilities to provide abortion services and shown that access to safe services remains a significant challenge for women in many settings.^21–23^ Nonetheless, there are some promising signs of progress; the MOH is currently in the process of recruiting additional health workers and strengthening primary healthcare. Furthermore, our study showed that the availability of family planning commodities has improved substantially since 2005; reducing unmet need for modern contraception will reduce the number of unintended pregnancies.^24^

As there are nearly 10 times as many clinical officers, three times as many midwives and nine times as many nurses relative to doctors in Central Province,^13^ provision of good quality TOP and PAC at the primary care level, and by midlevel providers, is essential to maximise access. There is currently ambiguity in the legal and regulatory framework, with the 1972 *Act* specifying a doctor, but the 2009 MOH guidelines stating that midlevel providers may be trained to provide first trimester procedures.^5 6^ In practice, training is not routinely provided to midlevel cadres, and those who receive training often to stop providing TOP due to stigma combined with the ambiguity and uncertainty. Allowing midlevel providers to provide abortion services has been shown to be of equal safety and effectiveness to provision by doctors in other low-income and middle-income settings^25 26^ and could be a relatively straightforward way to increase access without need for substantial additional resources.

Medical abortion (which in Zambia may be either using mifepristone and misoprostol in combination or misoprostol alone) was introduced to Zambia in 2009^14^ and is already the more frequently used method of both TOP and PAC. This is an important development since medical abortion is a safe and effective method of service delivery that can be provided discreetly and conveniently to women, with fewer physical barriers than surgical services (such as manual vacuum aspiration or dilation and evacuation, depending on gestation), and has the potential to reduce maternal mortality from unwanted pregnancy.^27 28^

Family planning availability improved between 2005 and 2016, particularly in hospitals and for long-acting reversible methods. This is consistent with a rapid increase in the modern contraceptive prevalence from 27% in 2007 to 41% in 2013–2014 among married women in Central Province.^29 30^ Emergency contraception availability increased from 11% to 33%, although availability is higher in hospitals than in health centres. Emergency contraception should be widely available at low-level facilities including health posts and with community health workers as well as pharmacy and drug stores so that it can be accessed as quickly as possible when needed.

A key strength of this study is that we conducted a census of all health facilities within Central Province, combined with the ability to link to population data with a questionnaire specifically designed to look at abortion signal functions. To our knowledge, this is the first time that this has been done in Zambia. Linking facility-based data to the community that the health system serves is a critical but underused component of health services planning. In addition, we achieved a high response rate covering both public and private facilities.

Our study also had some limitations. We were unable to compare PAC and TOP provision over time as the 2005 survey was designed for a more general purpose and there were important differences between the questionnaires used, which would make such an analysis unreliable. In particular, we believe that the 2005 survey overestimated TOP and PAC provision because of the general nature of the question wording.^17^ Second, we had to assume that the population is evenly distributed throughout wards for our geographic information systems analysis since we did not have data on distribution inside wards.

More facilities said they provided medical or surgical PAC than TOP in the past 12 months, despite the technical aspects of the procedures being similar. We are not able to discern the reason for this: it could be due to the current legal framework requiring three doctors to sign for a TOP but not for PAC, or it could be due to stigma. TOP in Zambia is highly stigmatised, and greater emphasis on values clarification activities for providers are likely to be necessary to improve abortion care and allow management to create a supportive environment for health workers to provide the essential reproductive health services recognised by the MOH. The political environment remains sensitive, and no health system exists in a vacuum outside of the wider social and political contexts. In order to address its maternal mortality problem, Zambia must ensure that a greater proportion of women have access to legal TOP and PAC services and services that provide choice in family planning method.
