# Supplementary figures and images for: Does supportive legislation guarantee access to pregnancy termination and postabortion care services? Findings from a facility census in Central Province, Zambia
Source: BMJ Glob Health. 2018 Sep 3;3(4):e000897. doi: 10.1136/bmjgh-2018-000897 (PMC6135439; doi:10.1136/bmjgh-2018-000897)

## Appendix 1: Map of facilities included in analysis

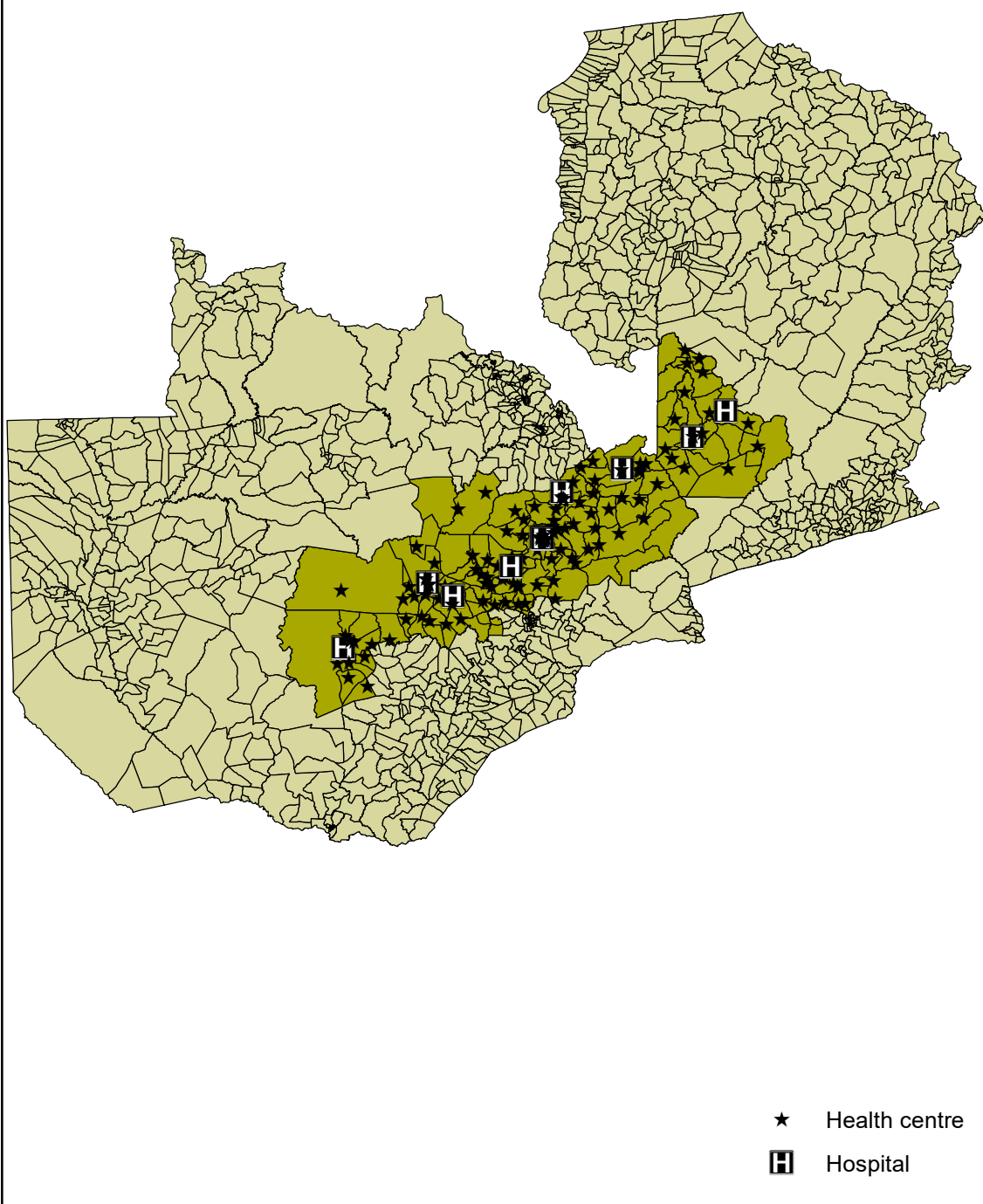

Supplement: Supplementary data [file bmjgh-2018-000897supp001.pdf]
